# Gut Microbiota and Colorectal Cancer: A Balance Between Risk and Protection

**DOI:** 10.3390/ijms26083733

**Published:** 2025-04-15

**Authors:** Vlad Alexandru Ionescu, Camelia Cristina Diaconu, Gina Gheorghe, Mara-Madalina Mihai, Carmen Cristina Diaconu, Marinela Bostan, Coralia Bleotu

**Affiliations:** 1 Faculty of Medicine, University of Medicine and Pharmacy Carol Davila Bucharest, 050474 Bucharest, Romania; vladalexandru.ionescu92@gmail.com (V.A.I.); gheorghe_gina2000@yahoo.com (G.G.); mara.mihai@umfcd.ro (M.-M.M.); 2 Internal Medicine Department, Clinical Emergency Hospital of Bucharest, 105402 Bucharest, Romania; 3 Academy of Romanian Scientists, 050085 Bucharest, Romania; cbleotu@yahoo.com; 4 Department of Oncologic Dermathology, “Elias” University Emergency Hospital, 010024 Bucharest, Romania; 5 Stefan S. Nicolau Institute of Virology, Romanian Academy, 030304 Bucharest, Romania; ccdiaconu@yahoo.com (C.C.D.); marinela.bostan@virology.ro (M.B.); 6 Department of Immunology, “Victor Babes” National Institute of Pathology, 050096 Bucharest, Romania; 7 Research Institute of the University of Bucharest (ICUB), University of Bucharest, 060023 Bucharest, Romania

**Keywords:** colorectal cancer, gut microbiota, risk factor, protective factor, microbial-derived metabolites

## Abstract

The gut microbiome, a complex community of microorganisms residing in the intestinal tract, plays a dual role in colorectal cancer (CRC) development, acting both as a contributing risk factor and as a protective element. This review explores the mechanisms by which gut microbiota contribute to CRC, emphasizing inflammation, oxidative stress, immune evasion, and the production of genotoxins and microbial metabolites. *Fusobacterium nucleatum*, *Escherichia coli* (pks+), and *Bacteroides fragilis* promote tumorigenesis by inducing chronic inflammation, generating reactive oxygen species, and producing virulence factors that damage host DNA. These microorganisms can also evade the antitumor immune response by suppressing cytotoxic T cell activity and increasing regulatory T cell populations. Additionally, microbial-derived metabolites such as secondary bile acids and trimethylamine-N-oxide (TMAO) have been linked to carcinogenic processes. Conversely, protective microbiota, including *Lactobacillus*, *Bifidobacterium*, and *Faecalibacterium prausnitzii*, contribute to intestinal homeostasis by producing short-chain fatty acids (SCFAs) like butyrate, which exhibit anti-inflammatory and anti-carcinogenic properties. These beneficial microbes enhance gut barrier integrity, modulate immune responses, and inhibit tumor cell proliferation. Understanding the dynamic interplay between pathogenic and protective microbiota is essential for developing microbiome-based interventions, such as probiotics, prebiotics, and fecal microbiota transplantation, to prevent or treat CRC. Future research should focus on identifying microbial biomarkers for early CRC detection and exploring personalized microbiome-targeted therapies. A deeper understanding of host–microbiota interactions may lead to innovative strategies for CRC management and improved patient outcomes.

## 1. Introduction

Currently, colorectal cancer (CRC) is the third most common type of cancer worldwide, accounting for 9.4% of all annually reported malignant diseases, and the second leading cause of cancer-related deaths, representing 9.3% of all such deaths [1]. CRC incidence and mortality rates vary significantly across geographic regions, reflecting disparities in risk factors, screening practices, and healthcare access [1]. According to the International Agency for Research on Cancer (IARC), the highest CRC incidence rates, ranging from 258.5 to 462.5 cases per 100,000 population, are observed in developed regions such as Australia, New Zealand, Europe (with the highest rates in Denmark and Norway), the United States, and Canada [1]. Conversely, the lowest CRC incidence rates, ranging from 35.9 to 112.4 cases per 100,000 population, are reported in developing regions such as Congo, Niger, Saudi Arabia, and India [1]. The geographic distribution of CRC-associated mortality rates closely follows the distribution of incidence rates [1].

**Figure 1:**
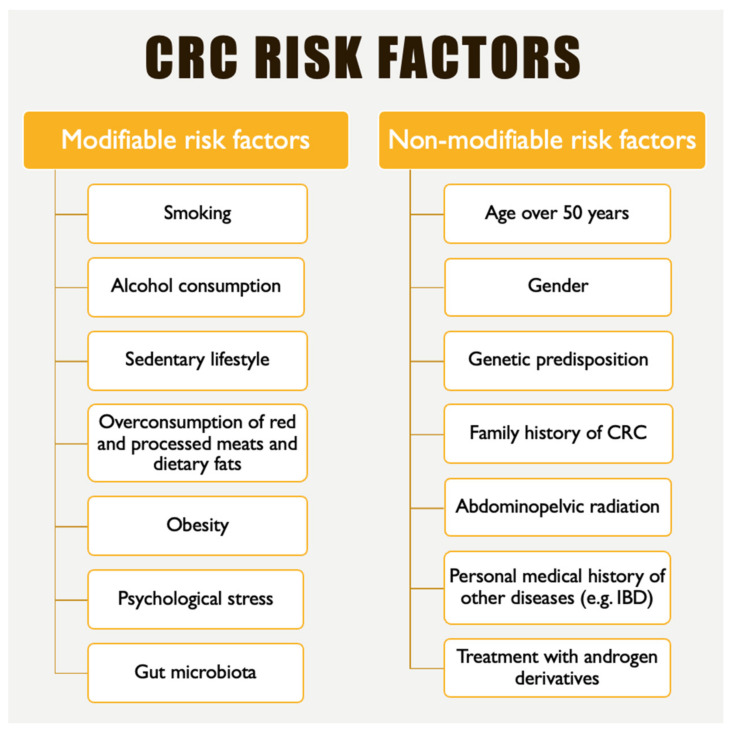
Colorectal cancer risk factors (IBD, inflammatory bowel disease) [3,4,5].

CRC incidence rates have steadily increased in countries undergoing economic transitions, including Eastern Europe, South America, Southeast Asia, and South–Central Asia [2]. The primary factors explaining this phenomenon are behavioral and dietary changes, specifically, higher consumption of animal-based foods and a sedentary lifestyle, which are associated with an increased prevalence of obesity [2]. Currently, there is robust evidence indicating that smoking, alcohol consumption, red or processed meat intake, and excess body fat increase the risk of CRC [2,3,4]. In contrast, the consumption of whole grains, dietary fiber, dairy products, and regular physical activity appears to have protective effects, particularly against colon cancer [2]. Figure 1 summarizes the key modifiable and non-modifiable risk factors associated with CRC [3,4,5].

The implementation of CRC screening programs has led to a decline in the incidence of this malignancy in developed countries [6]. However, a significant concern in recent years is the rising incidence of CRC in individuals under the age of 50, defined as early-onset colorectal cancer (EO-CRC), observed in both developed and developing countries [6,7,8]. For example, Yeo et al. recently reported an annual increase in EO-CRC incidence of approximately 1.4%, alongside a 3.1% yearly decline in CRC incidence among individuals over 50 years old (late-onset colorectal cancer—LO-CRC) [9]. This phenomenon suggests the profound influence of CRC risk factors during childhood and adolescence [2]. These risk factors include a sedentary lifestyle, the increasing prevalence of childhood obesity, and early antibiotic exposure, which may disrupt gut microbiota and contribute to CRC pathogenesis [2].

Emerging evidence highlights the critical role of gut microbiota, an essential component of the tumor microenvironment, in CRC development and progression. Gut dysbiosis—characterized by imbalances in bacterial, fungal, viral, and archaeal communities—has been strongly implicated in CRC pathogenesis through direct and indirect interactions with host epithelial and immune cells via metabolites, proteins, and macromolecules [10,11]. Additionally, the gut microbiota may play a significant role in the response of CRC patients to chemotherapy and immunotherapy and in the progression of CRC [10,11,12].

Clear evidence supporting gut dysbiosis as one of the pillars of CRC pathogenesis highlights the need for further research to elucidate the mechanisms underlying this causal relationship. Our study aims to present the most recent data emphasizing the role of gut dysbiosis in the development and progression of CRC. Understanding these mechanisms could lead to the development of personalized screening strategies for early diagnosis, the implementation of measures targeting gut microbiota modulation, and, consequently, the prevention of colorectal cancer or the improvement of responses to oncologic therapies.

Compared to prior systematic reviews and meta-analyses, this review offers a comprehensive and mechanistically detailed perspective by integrating both the pro-tumorigenic and protective roles of the gut microbiota in colorectal carcinogenesis. Furthermore, it incorporates recent advances concerning microbiota–immune checkpoint inhibitor (ICI) interactions and explores the therapeutic potential of microbial metabolites. By highlighting both harmful and beneficial microbial influences, alongside updated evidence on fecal microbiota transplantation (FMT) and microbial biomarkers, this review aims to provide a translational framework for the development of personalized microbiota-based interventions in colorectal cancer.

In preparing this narrative review, we conducted a comprehensive search of scientific publications from reputable academic journals, indexed in databases such as PubMed, Scopus, and Web of Science. Selection was based on the relevance to the topic, mechanistic depth, translational significance, and methodological quality. We included original articles, clinical studies, and high-impact reviews addressing both the protective and pro-tumorigenic roles of the gut microbiota in CRC. Where applicable, studies reporting conflicting or inconclusive findings were also considered and discussed in order to provide a balanced overview of the current state of research.

## 2. The Gut Microbiome

The human gastrointestinal tract hosts an extremely diverse and abundant microbial community [13]. It is estimated that the human gut contains over 100 trillion microorganisms belonging to more than 2000 different species, making it one of the most densely populated microbial habitats known [13,14]. This microbial community includes symbiotic, commensal, as well as pathogenic microorganisms [15]. Furthermore, the human gut microbiota encodes approximately 150 times more genes than the entire human genome [16]. These genes are largely shared among individuals, with approximately 99% being bacterial genes [16].

The terms “microbiota” and “microbiome” are often used interchangeably, although significant differences exist between them [17]. Microbiota refers to the entirety of living microorganisms within a defined environment, whereas the microbiome encompasses the total genomic content of microorganisms in a given environment, including not only the microbial community, but also microbial structural elements, metabolites, and environmental conditions [17]. From birth to approximately 3–4 years of age, an individual develops their primary resident microbiota [18]. However, defining the intestinal microbiome appears to require a longer period, ultimately resulting in a composition as unique as a fingerprint [19].

**Figure 2:**
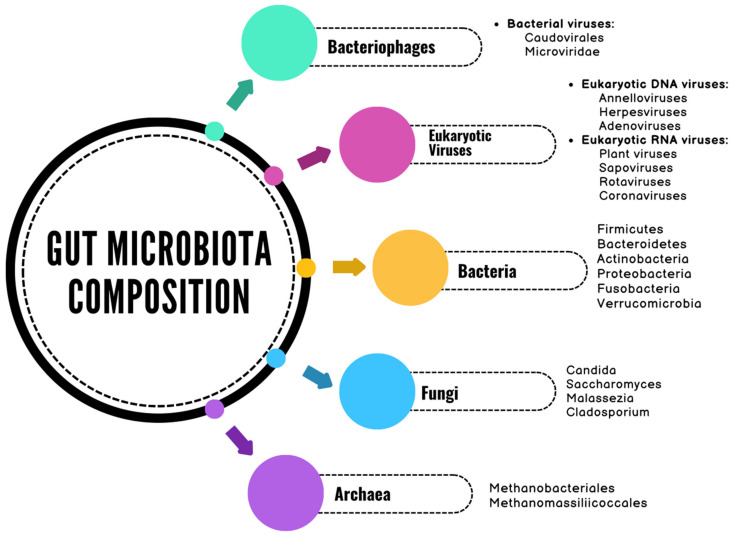
The composition of the gut microbiome (DNA, deoxyribonucleic acid; RNA, ribonucleic acid) [20,21,22].

The gut microbiota comprises a diverse array of microorganisms, including bacteria, viruses, bacteriophages, fungi, and archaea (Figure 2) [20,21,22]. The bacterial component of the gut microbiota is classified into six dominant phyla: Firmicutes, Bacteroidetes, Actinobacteria, Fusobacteria, Proteobacteria, and Verrucomicrobia [23]. Among these, Firmicutes and Bacteroidetes account for 90% of the gut microbiota composition [23]. The Firmicutes phylum consists of over 200 different genera, the most notable of which are Clostridioides, Lactobacillus, Bacillus, Enterococcus, and Ruminococcus [23]. The Bacteroidetes phylum also includes a large number of genera, with Bacteroides and Prevotella being predominant [23]. The Actinobacteria phylum is less abundant and is primarily represented by the Bifidobacterium genus [23].

The quantity of intestinal viruses in an adult is estimated to be approximately equal to that of intestinal bacteria, with over 10^12^ virus-like particles (VLPs) per individual, proportional to body size [24,25]. The human gut virome comprises eukaryotic viruses (<10%) and prokaryotic viruses (>90%) [21]. Eukaryotic viruses infect eukaryotic cells (primarily intestinal cells), while prokaryotic viruses, or bacteriophages, infect prokaryotic cells (mainly bacteria) [21]. Eukaryotic deoxyribonucleic acid (DNA) viruses, such as herpesviruses, adenoviruses, and anelloviruses, are largely latent and inactive under normal conditions, contributing to the regulation of host immunity [21]. Ribonucleic acid (RNA) viruses represent a significant etiological factor in gastrointestinal infections, often leading to inflammation, altered intestinal permeability, and digestive dysfunction. Among the most common enteric RNA viruses that affect the colon and gastrointestinal tract are norovirus (known as Norwalk Virus—a non-enveloped, positive-sense, single-stranded RNA virus belonging to the Caliciviridae family), rotavirus (a double-stranded RNA virus from the Reoviridae family), and astrovirus (a positive-sense, single-stranded RNA virus from the Astroviridae family) each with distinct epidemiological and clinical characteristics [26,27,28,29,30,31,32]. While norovirus remains the leading cause of acute gastroenteritis outbreaks, rotavirus predominantly affects infants and young children, and astrovirus is associated with milder, self-limiting infections.

Bacteriophages play an important role in the structure and function of the microbiome, constituting the majority of the intestinal virome [33]. Among them, bacteriophages of the order Caudovirales have been shown to be the most abundant in the human gut, accounting for over 90% of the intestinal virome content in adults [33,34,35].

Fungi represent another important component of the gut microbiome, influencing host immune responses by either attenuating or promoting local inflammatory reactions [36,37]. Among them, the most prevalent species in the human gastrointestinal tract is Candida albicans, a major inducer of T helper 17 (Th17) cells [37]. Other fungal species frequently identified in fecal samples include Saccharomyces and Malassezia [37,38,39]. However, there are questions regarding the natural residency of these two species in the intestinal tract, considering that Malassezia primarily colonizes the skin, while Saccharomyces spp. is commonly found in various beverages and foods [37].

The predominant archaea in the human gut are methanogens, which anaerobically reduce carbon dioxide to methane gas [40,41,42]. Among these, the most common species is Methanobrevibacter smithii, with a prevalence of approximately 95.7% [22,42].

The gut microbiome plays a crucial role in maintaining human body homeostasis by providing protection against pathogens, participating in digestion and metabolism, modulating insulin resistance and immune responses, controlling the proliferation and differentiation of epithelial cells, and influencing brain–gut communication, thereby impacting an individual’s neurological functions [43,44]. Protection against pathogens is achieved through competition for nutrients and adhesion sites at the intestinal mucosa, as well as the secretion of antimicrobial peptides [44]. A series of experiments conducted on germ-free animals have demonstrated the essential role of microbial colonization in the proper development of the immune system [45,46]. The absence of gut microbiota correlates with an underdeveloped intestinal mucosal immune system, including a reduced number of functional CD4+ and CD25+ regulatory T cells (Tregs) [45,46]. Additionally, the balance between pro-inflammatory effector T helper (Th) cells and Foxp3+ regulatory T (Treg) cells in the gut requires signals from intestinal bacteria [44]. For instance, the production of interleukin-10 (IL-10) via Tregs is essential for maintaining intestinal homeostasis, as it prevents excessive inflammation. Lactobacillus rhamnosus and Lactobacillus reuteri have been shown to induce IL-10 production via Tregs [47]. Furthermore, certain species of Lactobacillus have been associated with the increased production of interferon-gamma (IFN-γ) via T lymphocytes, macrophages, and dendritic cells, thereby stimulating protective immunity against intracellular pathogens [48,49]. On the other hand, interleukin-17A (IL-17A) is an important mediator of innate and adaptive immune responses, but can also contribute to inflammation and tissue damage [50]. Certain Bacteroides species, such as *Bacteroides fragilis*, have been associated with the induction of Treg differentiation from CD4+ T cells, thereby reducing IL-17A production [50]. Interleukin-22 (IL-22), produced by Th17 cells and type 3 innate lymphoid cells (ILC3), plays a role in defending against extracellular pathogens, as well as in epithelial regeneration and repair [51]. *Akkermansia muciniphila*, a constituent bacterium of the gut microbiota, has been shown to induce IL-22 production via ILC3 cells [51].

Furthermore, the gut microbiota produces a variety of bioactive compounds, such as short-chain fatty acids (SCFAs), which exhibit anti-inflammatory effects and contribute to maintaining the integrity of the intestinal barrier [43]. The main SCFAs are butyrate, acetate, and propionate [52]. Butyrate serves as the primary energy source for colonocytes and can regulate intestinal gluconeogenesis, providing beneficial effects on glucose and energy homeostasis [52,53]. Additionally, butyrate is essential for epithelial cell β-oxidation and the generation of a hypoxic state, which helps to prevent gut dysbiosis [52,53]. Propionate regulates gluconeogenesis and contributes to satiety induction through interaction with intestinal fatty acid receptors [52]. Acetate, an essential metabolite for the growth of other bacteria, also plays a role in cholesterol metabolism and lipogenesis [52]. Intestinal microbial enzymes contribute to bile acid metabolism, generating unconjugated bile acids that act as signaling molecules and metabolic regulators [52].

The composition of the gut microbiota has been shown to vary significantly between individuals [54,55,56]. Factors contributing to these differences include both genetic and environmental influences, such as geographic location, diet, and lifestyle [13]. For example, the Western lifestyle is associated with reduced bacterial diversity, the loss of certain species such as Prevotella, and a decline in the gut microbiota’s fiber-degrading functions [55]. In contrast, Mediterranean diets and plant-based diets have been shown to promote the production of beneficial bacterial metabolites, such as SCFAs, while simultaneously reducing concentrations of trimethylamine-N-oxide (TMAO), a compound associated with pro-inflammatory and proatherogenic properties [54]. Additionally, recent microbiome genome-wide association studies have demonstrated that certain genetic variants involved in immunity and intestinal architecture are associated with alterations in the composition of the gut microbiota [56]. Examples include variants in the lactase gene (LCT), fucosyltransferase 2 (FUT2), and nucleotide-binding oligomerization domain containing 2 (NOD2) [56]. Gut dysbiosis has been linked to endotoxemia, systemic inflammation, insulin resistance, and an increased risk of metabolic diseases, cardiovascular diseases, respiratory diseases, digestive diseases, autoimmune conditions, behavioral disorders, and cancer [20,57].

## 3. Mechanisms Linking Microbiota to Colorectal Cancer

Recent studies have demonstrated a strong association between gut dysbiosis and colorectal oncogenesis [10]. Advances in high-throughput microbiome sequencing and mass spectrometry have enabled the characterization of the oncogenic microbiome both within tumor formations and in non-tumoral regions of the colon [58]. However, variability in amplicon sequencing platforms, differences in sample types and collection time points, as well as discrepancies in analytical methods, pose challenges in achieving uniform conclusions [59,60]. This variability is further compounded by the heterogeneity of microbiome sequencing approaches, particularly between 16S ribosomal RNA (rRNA) gene sequencing and whole-metagenome shotgun sequencing. These methods differ substantially in terms of resolution, taxonomic coverage, and functional profiling capabilities. As a result, the comparability of findings across studies is often limited, and discrepancies in microbial composition or abundance may reflect methodological differences rather than true biological variation. In this review, we have taken into account the sequencing approach used in each referenced study and, where applicable, we have discussed how methodological heterogeneity may influence the interpretation of the results.

Gut dysbiosis refers to alterations in the composition of the gut microbiome, resulting from an imbalance between symbiotic and opportunistic microorganisms [61]. It is characterized by three main features: the overgrowth of pathogenic microorganisms, the loss of beneficial microbes, and a reduction in microbial diversity [62]. Various factors, including a Western diet, physical inactivity, obesity, smoking, antibiotic use, and aging, can promote the transition of the gut microbiome toward a pro-inflammatory profile [63,64]. With aging, there is a decline in CD4+ T lymphocytes and alterations in the gut microbiome, leading to the reduced ability of immune cells to suppress colonic inflammation [65,66]. Additionally, there is a decrease in butyrate-producing bacteria, which results in an increase in the intracolonic pH—a factor that, along with dysbiosis and chronic inflammation, contributes to CRC development [67].

The pathogenic link between gut dysbiosis and colorectal carcinogenesis involves several mechanisms, including the activation of pro-inflammatory signaling pathways, oxidative stress, and the modulation of antioxidant defense mechanisms. Additionally, dysbiosis contributes to immune evasion of antitumor responses, the production of genotoxins, virulence factors, and gut microbial metabolites, which further activate pro-oncogenic signaling pathways [68,69,70,71].

### 3.1. Microbiota-Mediated Inflammation and Immune Modulation

Under normal conditions, the gut microbiota does not trigger a significant inflammatory response, as the immune system has the ability to recognize commensal intestinal microorganisms [72]. A remarkable feature of the intestinal immune system is its ability to maintain immune tolerance toward a vast array of commensal microorganisms while simultaneously preserving an effective immune response against pathogenic infections and preventing the translocation of commensal microbes into the sterile environments of the body [73].

Gut dysbiosis plays a crucial role in inflammation, a process that promotes CRC development. The inflammatory mechanisms associated with colorectal oncogenesis include DNA damage induced by reactive oxygen species (ROS) and reactive nitrogen species (RNS), which are produced by dendritic cells, neutrophils, and macrophages, as well as the overproduction of cyclooxygenase-2 (COX-2) [69]. Furthermore, the invasion of the intestinal mucosa by pathogenic microorganisms leads to immune cell activation and the release of cytokines and growth factors that sustain the inflammatory process [69]. Chronic inflammation contributes to CRC development by stimulating epithelial cell proliferation, promoting angiogenesis, and inhibiting apoptosis [69]. Additionally, inflammatory factors can activate oncogenes or inactivate tumor suppressor genes, playing a key role in neoplastic progression [74].

Pattern-recognition receptors (PRRs), including toll-like receptors (TLRs) and nucleotide-binding oligomerization domain (NOD)-like receptors (NLRs), play a crucial role in detecting microorganism-associated molecular patterns (MAMPs), which are molecular structures specific to pathogenic microorganisms [75]. TLRs, expressed on macrophages and dendritic cells, are essential for microbial recognition and immune activation, particularly when the intestinal barrier is compromised [76]. There are two main TLR signaling pathways:

- The MyD88-dependent pathway, which involves the myeloid differentiation factor 88 (MyD88) adapter protein;
- The TRIF-dependent pathway, which relies on the TIR-domain-containing adapter-inducing interferon-β (TRIF) [77].

Salcedo et al. established a link between MyD88 inactivation and a diminished risk of CRC in a murine investigation [78]. Additionally, TLR-4 overexpression leads to the activation of NF-κB, resulting in COX-2 overproduction and an increased risk of CRC [79]. In contrast, TLR-2 expression plays a crucial role in maintaining intestinal homeostasis, and TLR-2 deficiency has been associated with a higher risk of CRC [80]. NLRs are intracellular receptors present in the cytoplasm of both immune and non-immune cells, and their activation triggers the synthesis of pro-inflammatory cytokines and autophagy [81]. Current data suggest significant differences in NLR signaling between tumor and non-tumor tissues in CRC patients [81].

Pro-inflammatory cytokines secreted by macrophages and T cells, such as interleukin-6 (IL-6) and tumor necrosis factor (TNF), induce the differentiation of Th17 cells, which exhibit a pro-inflammatory profile [82]. Elevated levels of interleukin-17 (IL-17) and interleukin-22 (IL-22), secreted by Th17 cells, have been correlated with reduced survival in CRC patients [82]. Furthermore, IL-6 promotes angiogenesis and activates the signal transducer and activator of the transcription 3 (STAT3) signaling pathway, serving as a negative prognostic marker for CRC patients [83].

In CRC patients, the cytolytic activity of natural killer (NK) cells against tumor cells is directly inhibited by the presence of *Fusobacterium nucleatum* in the tumor microenvironment [84]. This effect is partially mediated by the binding of the Fap2 protein of *F. nucleatum* to the human TIGIT receptor [84]. Additionally, elevated levels of *Fusobacterium nucleatum* are associated with a reduced density of CD3+ T cells, a cellular population linked to a more favorable clinical prognosis [85]. Conversely, intestinal commensal species of *Clostridium* utilize bile acids as signaling molecules to enhance the anti-tumor effects of hepatic CXCR6+ natural killer T (NKT) cells, influencing both metastatic progression and the development of primary liver tumors [86].

### 3.2. Oxidative Stress

Oxidative stress refers to an imbalance between oxidative molecules (reactive oxygen species [ROS] and reactive nitrogen species [RNS]) and antioxidant defense mechanisms. Recent studies have demonstrated a bidirectional correlation between the gut microbiota and oxidative stress [86]. The gut microbiota influences oxidative stress through metabolite synthesis, the regulation of antioxidant enzymes, and the maintenance of intestinal homeostasis [86]. Conversely, oxidative stress contributes to intestinal dysbiosis [80]. The gut microbiota is responsible for the production of metabolites such as short-chain fatty acids (SCFAs), including acetate, butyrate, and propionate, which have demonstrated antioxidant potential [86]. Additionally, the gut microbiota can modulate the production and activity of antioxidant enzymes [86]. For example, certain bacterial strains, such as *Lactobacillus plantarum* CCFM10, *Lactobacillus plantarum* CCFM1149, and *Bifidobacterium longum* CCFM752, have been shown to stimulate the production of antioxidant enzymes such as glutathione peroxidase, superoxide dismutase, and catalase [86].

Gut dysbiosis is associated with increased ROS production and compromised intestinal barrier function [87]. This disruption allows for the translocation of harmful substances and antigens across the intestinal epithelium, further amplifying oxidative stress and contributing to inflammation [88]. Oxidative stress activates NF-κB, leading to the overexpression of pro-inflammatory cytokines and anti-apoptotic signaling pathways [89,90]. Increased ROS production has been directly linked to CRC development and progression by promoting epithelial proliferation, angiogenesis, epithelial–mesenchymal transition (EMT), and the evasion of apoptotic mechanisms [91,92]. Additionally, certain bacterial species, such as *Bifidobacterium* and *Lactobacillus*, produce RNS, while Enterococcus faecalis generates hydroxyl radicals, contributing to CRC development by inducing point mutations and chromosomal breaks [69].

Although both pathogenic and protective bacterial strains influence oxidative balance, the current literature lacks direct comparative studies evaluating ROS production by these groups in standardized CRC models. Future research should aim to address this gap, as such studies would offer valuable insight into the specific redox-mediated contributions of different microbial taxa to colorectal carcinogenesis.

### 3.3. Evading the Antitumor Immune Response

A complex correlation between host immune system dysfunction and colorectal oncogenesis has been demonstrated in recent studies [93,94]. The immune system comprises innate immune cells, such as macrophages, neutrophils, dendritic cells, and NK cells, as well as adaptive immune cells, namely T and B lymphocytes [93]. These cells can exhibit both pro-oncogenic and anti-oncogenic functions [93].

One of the most notable characteristics of the gut microbiota is its role in promoting the maturation of immune cells [93]. This attribute is essential for the development and maintenance of the host’s immune system [95]. Certain commensal bacterial species residing in the colon, such as *Ruminococcus gnavus* and *Blautia producta*, have been shown to promote the activation of CD8+ T cells [96]. Consequently, these bacteria enhance the antitumor immune response by degrading tumor metabolites, such as hemolytic glycerophospholipids [96].

Intestinal dysbiosis promotes chronic inflammation, early T cell exhaustion, and the excessive stimulation of CD8+ T cells [97]. The consequence of this phenomenon is the suppression of antitumor immunity [97]. Another mechanism implicated in the adenoma–carcinoma sequence is the activation of the urea cycle, which is dependent on the absence of beneficial microorganisms with ureolytic capacity, such as *Bifidobacterium*, and on the overabundance of pathogenic bacteria that lack ureolytic function [98]. Urea can penetrate macrophages, inhibiting the binding of the phosphorylated signal transducer and activator of transcription 1 (p-STAT1) to the promoter region of spermidine/spermine N1-acetyltransferase 1 (SAT1) [98]. This leads to the polarization of macrophages toward a pro-tumor phenotype, characterized by the accumulation of polyamines [98]. Chen et al. demonstrated that treating a murine model with urea cycle inhibitors or supplementing it with beneficial *Bifidobacterium* species can mitigate urea-mediated colorectal oncogenesis [98].

On the other hand, tumors express neoantigens and develop mechanisms that allow them to evade immune detection, while also altering the composition of the gut microbiota [99]. Thus, the immune response in CRC is significantly influenced by the tumor’s neoantigen burden [99]. This is especially typical of tumors with deficiencies in the mismatch repair system, presenting as microsatellite instability [99]. Additionally, a subset of CRC demonstrates the evasion of immune surveillance, mediated by immune checkpoint regulators [99]. The immune response in CRC is further characterized by the infiltration of the stroma by tumor-associated macrophages and lymphocytes [99].

One mechanism that facilitates the evasion of the antitumor immune response is the upregulation of immune checkpoint inhibitors, such as programmed cell death protein 1 (PD-1) and cytotoxic T-lymphocyte antigen 4 (CTLA-4) [100]. The primary role of PD-1, a transmembrane protein, is to modulate immune responses by reducing inflammatory activity, thereby serving as an essential immune checkpoint [100]. Its ligand, PD-L1, is found on tumor cells, immune cells, and macrophages [100]. The interaction between PD-L1 and PD-1 suppresses the activation, proliferation, and antitumor capacity of CD8+ T cells, thereby facilitating immune evasion by the tumor [101]. Furthermore, the increased expression of CTLA-4 further impedes antitumor immune responses by competing with CD28 for B7 molecules on antigen-presenting cells, thus reducing T cell activation and proliferation [102]. Studies utilizing 16S and shotgun sequencing have identified several specific microbial taxa associated with a favorable response to PD-1/PD-L1 therapy [99]. Among these are *Prevotella*, *Ruminococcaceae,* and *Lachnospiraceae*, which were found in higher abundance in responders, suggesting their potential beneficial role in modulating the immune response [99]. Additionally, SCFA-producing bacteria, such as *Lactobacillus*, *Eubacterium*, and *Streptococcus*, have been positively associated with responses to PD-1/PD-L1 inhibitors [99]. In contrast, *Bacteroides* species were more abundant in non-responders, suggesting a negative impact on the efficacy of PD-1/PD-L1 therapies [99].

Additionally, the tumor microenvironment (TME) significantly influences tumor growth and distant metastasis by affecting processes such as cellular proliferation and immune response evasion [100]. The principal components of the TME include tumor-associated macrophages (TAMs), tumor-associated neutrophils (TANs), regulatory T cells, CD4+ T cells, and dendritic cells [100]. TAMs are divided into two main categories: M1-type TAMs, which possess antitumor properties, and M2-type TAMs, which exhibit immunosuppressive and protumoral characteristics [100]. M2-type TAMs accelerate tumor progression and invasion through the secretion of factors such as interleukin-10 (IL-10) and transforming growth factor beta (TGF-β) [100]. In CRC patients, intestinal dysbiosis promotes the increased expression of the secretory protein cathepsin K and M2-like macrophage polarization via tumor cells, thereby favoring CRC invasion and metastasis [103]. For example, it has been demonstrated that *Fusobacterium nucleatum* induces M2-like macrophage polarization and promotes CRC metastasis via the miR-1322/C-C motif ligand 20 (CCL20) axis [104]. Additionally, *Peptostreptococcus anaerobius* can increase the number of immune cells in the tumor, including TAMs, thereby promoting tumor progression [105]. In contrast, *Bifidobacterium adolescentis* inhibits colorectal carcinogenesis by recruiting and facilitating the infiltration of decorin+ macrophages via the activation of TLR2 and by regulating both primary human macrophages and M1 macrophages through the TLR2/yes-associated protein (YAP) axis [106]. Another hypothesis recently proposed by Dovrolis et al. is the existence of a power–law relationship between microbial diversity and tumor size [107]. This observation implies that larger tumors may harbor more diverse microbial communities, which could, in turn, influence tumor progression and therapeutic responses [107]. These findings highlight the importance of considering tumor-associated microbiota in the development of personalized treatment strategies for CRC.

Recent data suggest that the gut microbiome can modulate responses to anticancer immunotherapy [108,109,110]. One of the primary mechanisms by which the gut microbiota modulates antitumor immunity is through metabolites that can migrate from the intestinal tract, thereby influencing both local and systemic antitumor immune responses and enhancing the therapeutic efficacy of ICIs [110]. Furthermore, the gut microbiota can affect responses to CpG-oligodeoxynucleotide immunotherapy, adoptive T cell transfer immunotherapy, and cell-based immunotherapy [109]. For instance, *Akkermansia muciniphila*, *Faecalibacterium* spp., *Bacteroides fragilis*, and *Bifidobacterium* spp. have been associated with favorable antitumor immune responses in both preclinical tumor models and cancer patients [111]. In a recent study, Peng et al. analyzed fecal samples from patients with gastrointestinal cancer using 16S rRNA sequencing and reported an association between an increased *Prevotella/Bacteroides* ratio and a favorable response to Programmed Cell Death-1/Programmed Cell Death Ligand-1 (PD-1/PD-L1) immune checkpoint inhibitors [112]. A particular subgroup of responders exhibited a significantly higher abundance of *Prevotella*, *Lachnospiraceae*, and *Ruminococcaceae* [112]. Shotgun metagenomic analysis of the same samples further revealed that patients with differential treatment responses showed distinct metabolic pathway profiles, particularly those involved in nucleotide, lipid, carbohydrate, and short-chain fatty acid (SCFA) biosynthesis [112]. Another recent study has established a connection between a defined consortium of 11 bacterial strains and the induction of interferon-gamma-producing regulatory T cells within the intestinal mucosa [113]. These strains were found to exert their immunomodulatory effects in a synergistic manner, without eliciting inflammatory responses, through a mechanism dependent on CD103⁺ dendritic cells and major histocompatibility complex (MHC) class Ia molecules [113]. Furthermore, the colonization of murine models with this bacterial consortium resulted in a marked improvement in the efficacy of ICI therapy [113]. Elucidating the role of the gut microbiome in anticancer immunosurveillance and immunotherapy may provide promising perspectives for optimizing treatment responses in these patients.

### 3.4. Production of Genotoxins, Virulence Factors, and Intestinal Microbial Metabolites

Intestinal microbial metabolites can contribute to CRC progression by inducing DNA damage [114]. For example, certain strains of *Escherichia coli* produce toxins known as cyclomodulins, including colibactin, cytotoxic necrotizing factors, cytolethal distending toxins (CDTs), and cycle inhibiting factors [69]. Colibactin is a genotoxin that alkylates and crosslinks DNA, leading to double-strand breaks [114]. Colibactin has been positively associated with the development and progression of CRC [114]. CDTs have also been shown to be carcinogens that induce DNA damage [69]. Moreover, metabolites derived from strains of *Morganella morganii*, *Clostridium perfringens*, and *Clostridium ramosum* have been found to directly cause DNA damage [110]. Notably, the patterns of DNA damage induced by these metabolites differ from those induced by colibactin [110]. Certain species of *Morganella morganii* produce a distinct family of genotoxins termed indolimines [110].

**Figure 3:**
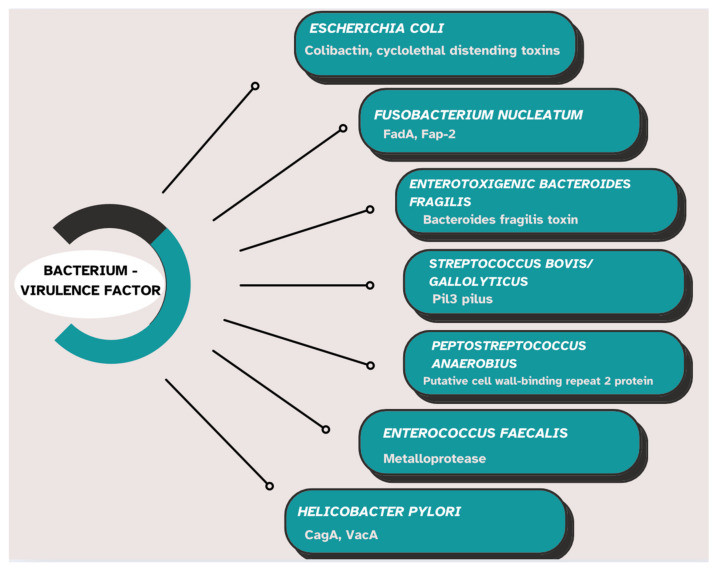
Virulence factors of the main intestinal bacteria implicated in colorectal oncogenesis (cytotoxin-associated gene A—CagA, vacuolating cytotoxin A—VacA) [69,111].

The virulence factors of the main intestinal bacteria implicated in colorectal oncogenesis are presented in Figure 3 [69,115].

*Fusobacterium nucleatum* utilizes the adhesin FadA for adhesion to and the invasion of epithelial cells. FadA stimulates the Wnt/β-catenin signaling pathway and increases cell permeability, thereby enabling the bacterium to traverse intercellular junctions [115]. The protein Fap2 can inhibit NK cells by binding to TIGIT, a receptor expressed on these cells [84,116].

Most strains of enterotoxigenic *Bacteroides fragilis* (ETBF) possess the BFT gene, which encodes the *Bacteroides fragilis* toxin (fragilysin or BFT) [117]. This zinc-dependent metalloprotease directly influences signaling pathways such as NF-κB, Wnt, and mitogen-activated protein kinase (MAPK), thereby stimulating cell proliferation and the production of pro-inflammatory mediators [118].

*Streptococcus gallolyticus* uses Pil3 for adhesion to and translocation across colonic epithelial cells [119]. *Peptostreptococcus anaerobius* interacts via its putative cell wall binding repeat 2 (PCWBR2) surface protein with α2/β1 integrins on colonic epithelial cells [105]. This interaction activates the PI3K-Akt signaling pathway, thereby stimulating inflammation and cellular proliferation [105]. *Enterococcus faecalis* generates reactive oxygen species (ROS) and extracellular superoxide, which can induce DNA damage in colonic epithelial cells [120]. Furthermore, *Enterococcus faecalis* produces a metalloprotease that promotes inflammation and adversely affects the intestinal epithelial barrier [121].

Both in patients with adenomas and those with colorectal adenocarcinomas, increased colonization of the colonic mucosa with *Helicobacter pylori* has been identified [122]. The pathogenic factors of *Helicobacter pylori*, such as cytotoxin-associated gene A (CagA) and vacuolating cytotoxin A (VacA), can activate pro-inflammatory signaling pathways, thereby stimulating cellular proliferation [123].

According to data from the past decades, certain microbial taxa exert specific effects on gene expression, thereby contributing to colorectal oncogenesis (Table 1) [99].

Currently, it is estimated that approximately 50% of plasma metabolites originate from bacterial sources [124]. Specific categories of intestinal microbial metabolites include SCFAs, secondary bile acids, indoles, polyphenols, polyamines, methylamines, vitamins, and other compounds [125,126]. SCFA concentrations vary along the intestinal tract, reaching their highest levels in the cecum and proximal colon [127]. Their concentrations decrease in the distal colon due to absorption by colonic epithelial cells [127]. Consequently, only 5–10% of the total SCFAs remain unabsorbed and can be detected in fecal samples [127]. The majority of acetate and propionate enter systemic circulation, whereas butyrate plays a protective role in CRC [127]. The direct interaction of SCFAs with the colonic epithelium has recently gained attention due to its presumed involvement in colorectal oncogenesis [127]. In CRC, butyrate inhibits tumorigenesis by directly suppressing histone deacetylase (HDAC) activity, thereby modulating the expression of tumor suppressor genes [128]. Additionally, butyrate is involved in the metabolic reprogramming of cancer cells and the activation of G-protein-coupled receptor-dependent signaling pathways, mechanisms that lead to anti-inflammatory responses and cancer cell apoptosis [128]. Moreover, in CRC cell lines, butyrate has been shown to induce the expression of cell cycle regulators *p21* and *p27*, as well as pro-apoptotic genes such as *FAS*, through the stimulation of histone acetylation, thereby inhibiting proliferation and promoting apoptosis [129].

Secondary bile acids, particularly deoxycholic acid (DCA), contribute to colorectal oncogenesis. The primary bacterial genera involved in the biosynthesis of secondary bile acids include *Bacteroides, Eubacterium, Bifidobacterium, Lactobacillus*, and *Clostridium* [130,131]. A multi-omics study reported increased fecal concentrations of DCA and elevated levels of 7α-dehydroxylating bacteria in populations at high risk of CRC [125]. Another study demonstrated a correlation between elevated bile acid levels and the abundance of *Bilophila wadsworthia* [132]. DCA exerts multiple pathogenic effects on the colonic epithelium, including the disruption of the cell membrane, induction of oxidative DNA damage, and activation of the NF-κB signaling pathway [133]. Additionally, DCA antagonizes the function of the intestinal Farnesoid X receptor (FXR), thereby promoting cellular proliferation and the expansion of *Lgr5+* cancer stem cells [134]. In contrast, ursodeoxycholic acid (UDCA) and tauroursodeoxycholic acid (TUDCA) inhibit colorectal cancer development [135,136]. UDCA regulates intracellular reactive oxygen species (ROS) production and suppresses cell cycle progression in tumor cells, whereas TUDCA inhibits NF-κB signaling [135,136].

The intestinal tract contains high concentrations of polyamines, primarily putrescine, spermine, and spermidine, which originate from dietary intake or are biosynthesized by gut bacteria [137]. Polyamines are involved in numerous biological processes, including cellular proliferation, differentiation, and immune cell activation [138]. Dysregulation of polyamine metabolism, either by the host or the gut microbiota, may contribute to CRC development. The catabolism of polyamines induced by pathogenic species generates a series of reactive toxic metabolites that can damage DNA, proteins, and other cellular components. For example, enterotoxigenic *Bacteroides fragilis* produces the enzyme spermine oxidase, which catalyzes the conversion of spermine to spermidine, generating hydrogen peroxide as a byproduct [139]. This reaction promotes intracellular oxidative stress, leading to DNA damage and accelerating carcinogenesis [139]. Furthermore, polyamines activate oncogenic signaling pathways such as pAKT and β-catenin and suppress antitumor immune responses [140,141].

## 4. Pathogenic Microbiota Associated with Colorectal Cancer

Alterations in the composition of the intestinal microbiota are associated with intestinal barrier dysfunction, increased intestinal permeability, elevated plasma lipopolysaccharide concentrations, and chronic low-grade inflammation, all of which facilitate tumorigenesis [142]. The most significant bacterial species implicated in colorectal carcinogenesis include *Fusobacterium nucleatum*, *Bacteroides fragilis*, *Streptococcus gallolyticus*, *Enterococcus faecalis*, *Escherichia coli*, *Helicobacter pylori*, and *Clostridium septicum* [94,143]. However, no bacterial species has yet demonstrated sufficient virulence to independently cause the disease [144]. This may suggest the existence of specific combinations of microorganisms that act synergistically to modify the intestinal microenvironment and initiate carcinogenesis [144]. Moreover, microbial signatures characteristic of CRC include not only an altered bacterial composition, but also a dysregulated virome [140]. However, data regarding changes in the intestinal virome in CRC remain limited and contradictory [144]. For example, Nakatsu et al. reported an increased abundance of *Orthobunyavirus* in fecal samples from CRC patients, along with a rise in the number of bacteriophages known to infect Gram-negative bacteria associated with this malignancy [145]. Other authors have suggested an indirect role of bacteriophages in colorectal oncogenesis [146]. Additionally, *Enterobacteria* phages, which predominate in healthy individuals, are significantly reduced in CRC patients [95].

*Fusobacterium nucleatum* is a bacterium typically found in the oral cavity, rarely colonizing the lower gastrointestinal tract of healthy individuals [147]. However, colorectal tumors exhibit an increased abundance of this bacterium, which has been associated with disease recurrence, distant metastases, and poor prognosis [147]. Strains isolated from tumors predominantly belong to *Fusobacterium nucleatum* subspecies *animalis* (Fna) [147]. Zepeda-Rivera et al. demonstrated the subdivision of this subspecies into two distinct clades, Fna C1 and Fna C2 [147]. Among these, only Fna C2 was found to dominate the colorectal tumor niche [147]. Furthermore, the abundance of *Fusobacterium nucleatum* varies significantly among CRC patients, suggesting the involvement of additional, individual-specific effectors [148]. A recent study demonstrated the preferential enrichment of this bacterium in tumor tissues harboring the KRAS p.G12D mutation [148]. Additionally, *Parabacteroides distasonis* competes with *Fusobacterium nucleatum* in colorectal tumors carrying the KRAS mutation [148]. Oral administration of *Parabacteroides distasonis* in mice attenuates *Fusobacterium nucleatum*-dependent CRC progression [148].

Another mechanism by which *Fusobacterium nucleatum* contributes to colorectal oncogenesis is through its binding to DHX15, an RNA helicase family protein expressed on colorectal tumor cells, leading to the activation of the ERK/STAT3 signaling pathway [148]. Moreover, *Fusobacterium nucleatum* can directly increase DNA methyltransferase activity, resulting in the hypermethylation of tumor suppressor genes [149]. This process contributes to a high microsatellite instability (MSI-H) phenotype and the development of a CpG island methylator phenotype (CIMP) in CRC [149]. These epigenetic changes may be secondary to the involvement of *Fusobacterium nucleatum* in regulating inflammatory cytokine production and reactive oxygen species (ROS), which can influence DNA methylation patterns [150]. Additionally, *Fusobacterium nucleatum* disrupts the Chk2 signaling pathway, leading to impaired cell cycle regulation and defective DNA repair mechanisms [150]. Furthermore, it promotes chemoresistance by interfering with autophagy, a crucial cellular process essential for survival under stress conditions [151].

**Figure 4:**
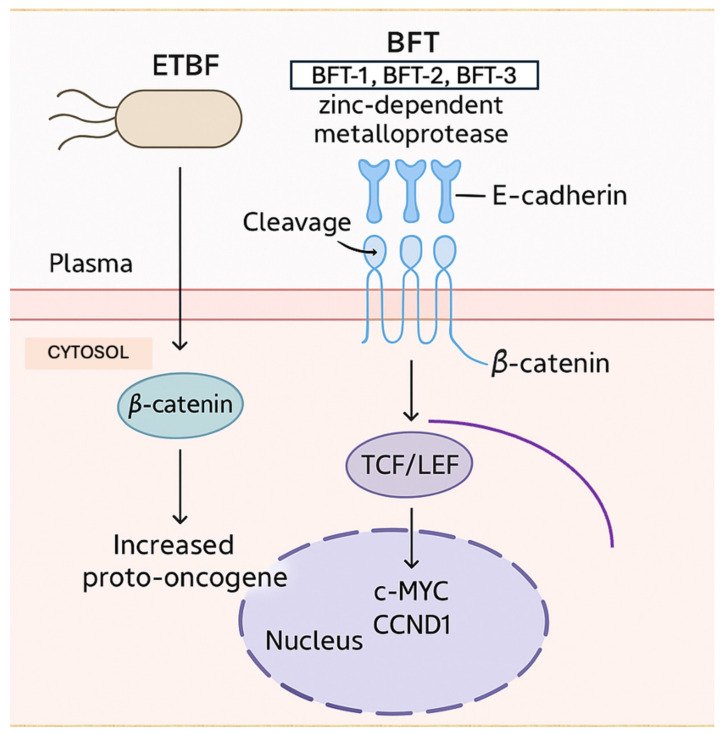
Oncogenic mechanism of Enterotoxigenic Bacteroides fragilis (ETBF) via E-cadherin cleavage and β-catenin-mediated activation of proto-oncogenes (transcription factor/lymphoid enhancer-binding factor [TCF/LEF], cellular myelocytomatosis oncogene (*c-MYC),* and cyclin D1 [CCND1]) [152].

*Enterotoxigenic Bacteroides fragilis (ETBF)* produces a zinc-dependent metalloprotease, BFT, with three isoforms: BFT-1, BFT-2, and BFT-3 [152]. This toxin exerts its oncogenic effect by cleaving the extracellular domain of E-cadherin, a tumor suppressor protein essential for maintaining epithelial cell integrity [152]. The cleavage of E-cadherin leads to the accumulation of β-catenin in the cytosol and its subsequent translocation into the nucleus [152]. At the nuclear level, β-catenin binds to the transcription factor/lymphoid enhancer-binding factor (TCF/LEF), thereby increasing the transcription of proto-oncogenes cellular myelocytomatosis (c-MYC) and cyclin D1 (CCND1) (Figure 4) [152]. Furthermore, a correlation has been demonstrated between elevated ETBF levels and the increased expression of axis inhibitor (AXIN) and catenin beta 1 (CTNNB1) in tumor tissues [152]. BFT may also contribute to oncogenesis by upregulating the MAPK and WNT signaling pathways and activating pro-inflammatory cytokines such as IL-8 [152].

*Streptococcus gallolyticus* subspecies *gallolyticus* (Sgg) stimulates cell proliferation and promotes the development of colorectal cancer (CRC) [153]. Additionally, phenotypic heterogeneity among *Sgg* strains has been demonstrated regarding their ability to induce cell proliferation [153]. For instance, strain TX20005 exhibits this property, whereas strain ATCC_43143 does not [153]. Deletion of the *Sgg* pathogenicity-associated region (*SPAR*) locus leads to a reduction in *Sgg* colonization in the colon, as well as a decreased ability of this bacterium to adhere to epithelial cells, stimulate cell proliferation, and promote CRC development [153]. Bacteremia with *Sgg* increases the risk of colonic adenomas and adenocarcinomas by 60% [154]. Furthermore, 45% of patients with *Sgg*-induced endocarditis develop colorectal tumors within five years of infection diagnosis [155]. This bacterium is implicated in the activation of pro-oncogenic signaling pathways, including *c-Myc*, *Wnt/β-catenin*, and *proliferating cell nuclear antigen (PCNA)* [155]. *β-catenin* is essential for *Sgg*-induced cell proliferation [156]. The inhibition of *β-catenin* expression in responsive cells abolishes *Sgg*’s effect on cell proliferation [156].

**Figure 5:**
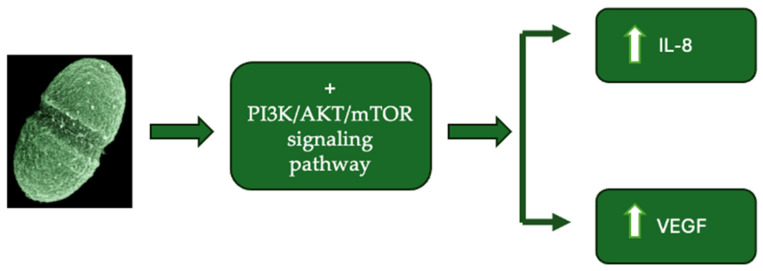
*Enterococcus faecalis* activates the PI3K/AKT/mTOR signaling pathway, upregulating the expression of interleukin-8 (IL-8) and vascular endothelial growth factor (VEGF), mechanisms which underlie its pro-proliferative and pro-angiogenic effects [157].

*Enterococcus faecalis* (*Efa*) promotes CRC development primarily through its metabolite biliverdin, which exerts proliferative and angiogenic effects on colorectal tumor cells via the activation of the PI3K/AKT/mTOR signaling pathway [157]. *Efa* upregulates the expression of interleukin-8 (IL-8) and vascular endothelial growth factor (VEGF) (Figure 5) [157]. IL-8 has multiple pro-oncogenic functions, including altering the composition of the tumor microenvironment, promoting the transformation of tumor cells into a mesenchymal or migratory phenotype, enhancing tumor angiogenesis, and recruiting a high number of immunosuppressive cells to the tumor site [157]. VEGF induces angiogenesis by activating signaling pathways involved in increasing vascular permeability, as well as promoting endothelial cell growth, migration, and differentiation [157]. Moreover, *Efa* produces extracellular superoxide, which leads to DNA damage and genomic instability in intestinal epithelial cells [158]. Another mechanism through which this microorganism contributes to oncogenesis involves the inhibition of the protective TGF-β/Smad signaling pathway [159].

*Escherichia coli* can be classified into four phylogroups: A, B1, B2, and D [160]. Virulent *E. coli* strains predominantly belong to phylogroup B2, which has been implicated in colorectal oncogenesis [160]. The in vitro incubation of *E. coli* B2 phylogroup strains with various epithelial cell lines leads to cell cycle arrest, cell elongation, and entry into senescence [160]. This effect is attributed to a group of compounds known as cyclomodulins, which induce double-strand DNA breaks in target cells [160]. Cyclomodulins produced by *E. coli* include colibactin, cytotoxic necrotizing factor, cytolethal distending toxin, and cycle inhibiting factor [160]. Colibactin activates the Wnt signaling pathway by modifying Wnt proteins and inhibiting β-catenin degradation, ultimately causing DNA damage [161]. These effects result in transient cell cycle arrest in the G2-M phases, followed by cell death [161]. In this manner, infected cells may survive even after incomplete DNA repair, leading to an increased mutation rate and carcinogenesis [161].

Beyond its well-established role in gastric carcinogenesis, recent studies have demonstrated an association between *Helicobacter pylori (H. pylori)* infection and CRC [162]. A large-scale study involving 812,736 individuals reported that *H. pylori* infection is associated with an 18% increased risk of CRC and a 12% increased risk of CRC-related mortality [162]. The potential pathogenic mechanisms underlying the link between *H. pylori* infection and colorectal oncogenesis include the disruption of cellular proliferation by the VacA toxin, association with a specific immune profile characterized by a reduction in regulatory T cells, activation of the pro-oncogenic STAT3 signaling pathway, and loss of goblet cells [163].

Regarding the relationship between *Clostridium septicum* and CRC, the current evidence suggests that this bacterium does not initiate carcinogenesis, but rather promotes local tumor proliferation and the hematogenous dissemination of malignant cells [164]. The hypoxic and acidic tumor microenvironment facilitates the germination of *Clostridium septicum* spores and bacterial proliferation, with alpha toxin production contributing to mucosal ulceration and the systemic dissemination of malignant cells [164]. Furthermore, histological studies have demonstrated a paucity of leukocytes within areas of *Clostridium septicum*-induced myonecrosis, a phenomenon attributed to the alpha toxin’s selective ability to induce neutrophil apoptosis, thereby impairing the host immune response [164]. Through this mechanism, both bacterial growth and tumor progression are promoted [165].

## 5. Protective Microbiota in Colorectal Cancer

The gut microbiome has recently been recognized as an organ due to its involvement in maintaining systemic homeostasis, influencing metabolism, inflammatory responses, intestinal barrier integrity, and the neurological, endocrine, and immune systems [166,167]. An intact intestinal barrier consists of tight junctions, a mucus layer, and antimicrobial peptides such as defensins, cytokines, and immunoglobulin A (IgA) [11,168]. Moreover, the gut microbiota is involved in the biosynthesis of biologically active molecules, including vitamins, SCFAs, amino acids, and lipids [11,168].

The gut microbiome plays a crucial role in colorectal oncogenesis, both through direct interaction with enterocytes and by influencing cellular metabolism and immune responses [169]. Investigations over the decades have revealed alterations in gut microbiota composition and a decrease in its diversity in patients with CRC [169]. Under conditions of mild colonic inflammation, certain microbial species exert a protective effect against colorectal oncogenesis [166]. This mechanism involves the microbiota-mediated suppression of the long non-coding RNA (lncRNA) Snhg9, which promotes tumor growth by inhibiting the tumor suppressor p53 [166]. Consequently, intestinal dysbiosis may lead to increased Snhg9 expression and accelerate CRC progression [166].

Among the bacterial species with protective effects against CRC are Lactobacillus, Bifidobacterium, Faecali bacterium prausnitzii, Roseburia spp., and Akkermansia muciniphila (Table 2) [170,171,172,173,174]. Additionally, Faecalibaculum rodentium and *Streptococcus thermophilus* have been suggested as potentially beneficial, but further studies are needed to confirm their role in colorectal carcinogenesis (Table 2) [170].

*Lactobacillus* species are frequently used as dietary supplements, and their role in protecting against CRC was initially investigated in mice [170]. The beneficial effects of *Lactobacillus rhamnosus*, *Lactobacillus fermentum*, and *Lactobacillus acidophilus* against this malignancy have been demonstrated [170]. *Lactobacillus sakei* CVL-001 enhances the therapeutic efficacy of mesenchymal stem cells (MSCs). It accomplishes this by initiating an immunoregulatory phenotype by activating the STAT3 signaling pathway and increasing the secretion of IL-10 [174].”

Lactobacilli metabolize carbohydrates to produce lactic acid, making them the most abundant genus within the lactic acid bacteria (LAB) group [175]. This characteristic allows them to modify the intestinal lumen pH, inhibiting the growth of certain bacteria such as *Bacteroides* and damaging the outer membrane of Gram-negative bacteria, including *Pseudomonas aeruginosa*, *Escherichia coli* O157:H7, and *Salmonella enterica* [176]. Additionally, *Lactobacillus* strains produce short-chain fatty acids (SCFAs), which have been shown to enhance transepithelial electrical resistance and stimulate the formation of tight junctions between intestinal epithelial cells [177]. *Lactobacillus plantarum* strains produce multiple bacteriocins with antimicrobial activity against pathogens such as *Listeria monocytogenes*, as well as against food spoilage bacteria [178].

Lactobacillus species have been shown to reduce the levels of carcinogenic agents such as N-nitrosamines, heterocyclic amines, and secondary bile acids [178,179]. Lactic acid-producing bacteria can directly degrade N-nitrosodimethylamine and enhance the ability to eliminate nitrites from fermented foods [179]. Moreover, *Lactobacillus* species possess bile salt hydrolase activity, which enables them to deconjugate bile acids, thereby reducing the formation of harmful secondary bile acids [180].

*Bifidobacterium* species have demonstrated anticancer activity in both in vivo and in vitro studies [181,182]. *Bifidobacterium longum* enhances interactions between NK cells and dendritic cells, stimulating IFN-γ production by NK cells, thereby contributing to immune modulation [182]. Extracts of *Bifidobacterium adolescentis* have been shown to inhibit the growth of CRC cell lines by reducing the activity of β-glucuronidase, tryptophanase, and urease [183]. Furthermore, the ingestion of *Bifidobacterium longum* by CRC patients has been linked to modifications in the gut microbiota, resulting in enhanced microbial diversity [183]. This enhances the integrity of tight junctions and diminishes epithelial permeability, which is essential for preserving intestinal barrier function [183].

*Faecalibacterium prausnitzii* is one of the main butyrate producers in the intestine [184]. Butyrate can reduce intestinal mucosal inflammation by inhibiting the NF-κB transcription factor and interferon-gamma (IFN-γ), while upregulating peroxisome proliferator-activated receptor gamma (PPARγ) [184]. Additionally, this species exhibits anti-inflammatory properties by inducing a tolerogenic cytokine profile, characterized by the very low secretion of pro-inflammatory cytokines such as IL-12 and IFN-γ, and increased secretion of anti-inflammatory cytokines such as IL-10 [184]. In a murine model, the administration of *Faecalibacterium prausnitzii* significantly reduced azoxymethane-induced aberrant crypt foci formation in the colon [171]. Furthermore, this microorganism decreased lipid peroxidation levels in colonic tissues, further supporting its protective role in colorectal carcinogenesis [171].

*Akkermansia muciniphila* plays a crucial role in maintaining the integrity and dynamics of the intestinal mucus layer [185]. It degrades mucins, utilizing them as sources of nitrogen, carbon, and energy, while producing SCFAs and oligosaccharides [185]. *A. muciniphila* selectively recognizes the unsialylated LacNAc disaccharide (Galβ1-4GlcNAcβ1-R) on core 2 and core 3 O-glycans [186]. This disaccharide epitope is abundantly found on human colonic mucins, capped by sialic acid [186]. Additionally, *A. muciniphila* can expose this epitope through its endogenous neuraminidase activity [186]. Furthermore, it attenuates the inflammatory response and reduces intestinal permeability by producing SCFAs, inhibiting histone deacetylases (HDACs), and activating G-protein-coupled receptors (GPRs) [185]. A study by Derosa et al. utilizing shotgun metagenomic sequencing demonstrated a correlation between the presence of *A. muciniphila* in the gut and improved response rates to PD-L1 inhibitor therapy, independent of PD-L1 expression levels [186]. Additionally, intestinal colonization by *A. muciniphila* was associated with increased gut microbiome diversity and a more inflammatory tumor microenvironment, suggesting a potential role in modulating immunotherapy outcomes [186].

Studies have shown a reduction in the presence of *Roseburia intestinalis* in fecal samples of CRC patients compared to healthy subjects [187]. *Roseburia intestinalis* contributes to restoring intestinal barrier function, improves intestinal permeability, and enhances the expression of tight junction proteins [187]. Additionally, the butyrate produced by this bacterium plays a crucial role in suppressing tumor growth [187]. Moreover, *Roseburia intestinalis* and butyrate significantly enhanced the efficacy of anti-PD-1 therapy in mice bearing MSI-low tumors [187]. The underlying mechanism involves the direct binding of butyrate to the TLR5 on CD8+ T cells, the activation of NF-κB-dependent signaling, and the amplification of the antitumor immune response [187].

Data regarding the protective effects of *Faecalibaculum rodentium* and *Streptococcus thermophilus* against CRC remain limited. Both *Faecalibaculum rodentium* and its human homolog, *Holdemanella biformis*, are involved in the production of SCFAs, which regulate tumor cell proliferation and protein acetylation by suppressing calcineurin and NFATc3 activation [188]. The β-galactosidase secreted by *Streptococcus thermophilus* has been shown to inhibit cell proliferation and cell cycle progression while promoting tumor cell apoptosis [189]. Furthermore, β-galactosidase can increase the abundance of *Lactobacillus* and *Bifidobacterium*, suggesting a synergistic effect between these bacterial species [189]. Additionally, *Streptococcus thermophilus* may suppress tumor growth through the release of folate, a crucial element in cellular metabolism, DNA replication and repair, methylation, and nucleotide synthesis [190].

Recent studies have demonstrated the efficacy of FMT and probiotics in inhibiting the development of colorectal cancer (CRC) through the restoration of the gut microbiota balance, amelioration of excessive intestinal inflammation, and enhancement of antitumor immune responses (Table 3) [191,192,193,194,195,196,197].

The transplantation of healthy microbiota has been shown to suppress tumor progression, being associated with a reduction in both the number and size of tumor lesions, as well as a significant prolongation of overall survival [191]. Histological analysis in CRC mice receiving FMT revealed the increased infiltration of cytotoxic immune cells, including CD8+ T lymphocytes and CD49b+ NK cells, which are directly involved in tumor cell destruction [191]. Concurrently, a reduction in Foxp3+ regulatory T cells, commonly observed in CRC, was also noted [191]. Furthermore, FMT modulated the expression of inflammatory cytokines, leading to decreased levels of IL-1α, IL-6, IL-12α, IL-12β, and IL-17α, alongside elevated levels of IL-10 [191]. Additionally, FMT influenced oncogenic signaling pathways by downregulating STAT3 and TGF-β expression, while upregulating TNF-α, CXCR4, and IFN-γ, thereby contributing to the amplification of antitumor immune responses [191]. Moreover, FMT could enhance the efficacy of immunotherapy by modulating the interactions between immune and tumor cells, as well as by modifying microbial metabolites, thereby exerting a direct impact on the tumor microenvironment [192]. Thus, FMT may represent a promising adjuvant therapy to ICIs, contributing both to enhancing their efficacy and reducing the heterogeneity of therapeutic responses [192]. Nevertheless, many questions remain regarding the optimal composition of the gut microbiota capable of amplifying ICI outcomes, as well as the long-term safety of FMT [192].

Despite its therapeutic potential, FMT raises important safety considerations. Adverse effects such as gastrointestinal disturbances, bacteremia, and the transmission of antibiotic-resistant or opportunistic organisms have been reported [198,199]. To mitigate these risks, strict donor screening protocols—including infectious screening and medical history review—are critical [198,199]. In addition, patient selection remains an open question. The current evidence suggests that host immune status, prior exposure to antibiotics or immunosuppressants, and individual microbiota composition may influence both safety and therapeutic outcomes [198,199]. Further clinical trials are necessary to establish long-term safety, define optimal FMT protocols, and identify which CRC patients are most likely to benefit from this approach, particularly in combination with ICIs.

Another clinical application of gut microbiome analysis may lie in its contribution to the early diagnosis of CRC. Rezasoltani et al. recently reported significant differences in the microbial profiles of saliva and fecal samples between CRC patients and healthy individuals [200]. Specifically, the authors identified an increased abundance of *Granulicatella adiacens*, *Calothrix parietina*, *Rothia mucilaginosa*, and *Rothia dentocariosa* in the saliva of CRC patients, with these species being absent in the saliva of healthy controls [200]. Additionally, the families *Prevotellaceae* and *Lachnospiraceae* were significantly more abundant in the fecal samples of CRC patients compared to healthy subjects [194]. In contrast, *Akkermansia muciniphila* was found to be more abundant in the healthy cohort [200]. These findings are supported by another study, which concluded that both the salivary and intestinal microbiomes may serve as potential biomarkers for the early detection of CRC [201].

## 6. Concluding Remarks and Future Direction

The gut microbiome and especially microbial signatures represent an essential source of biomarkers for CRC diagnosis and prognosis. The increased presence of *Fusobacterium nucleatum*, toxigenic *Bacteroides fragilis*, and colibactin-producing *Escherichia coli* is associated with an increased risk of colorectal carcinogenesis. Microbiota analysis of fecal samples offers a non-invasive screening method with high sensitivity and specificity, which can differentiate between CRC stages. The microbiota composition can predict the response to treatment, with microbial profiles changing during therapy correlated with the efficacy of the treatment, a mechanism that helps to stratify patients by identifying patients at high risk of recurrence. The development of standardized methods for assessing the microbiota and their integration into clinical practice could significantly improve the early detection and personalization of treatment for patients with colorectal cancer. Given the rapid advances in microbiota analysis technologies, this emerging field could strongly influence the future of personalized oncology. Combining microbiota analysis with artificial intelligence and advanced machine learning algorithms could facilitate the identification of more accurate predictive models, accelerating the implementation of these methods in the clinic.

On the other hand, personalizing therapy by manipulating the microbiome through diet, probiotics, or fecal microbiota transplantation can optimize therapeutic outcomes by optimizing the immune response and reducing inflammation. Future research could explore the role of the microbiota in modulating the immune response and in the development of personalized therapies based on the interaction between the microbiome and the host, offering new perspectives in the treatment of CRC.

Despite the promising potential of microbiota-targeted therapies, several key barriers hinder their integration into clinical practice. These include the high inter-individual variability of the gut microbiome, which limits the generalizability of findings, and the lack of standardized protocols for microbiota sampling, analysis, and interpretation. Additionally, our current understanding of host–microbiota–immune interactions remains incomplete, complicating the identification of reliable microbial biomarkers and therapeutic targets. Safety concerns regarding microbiota interventions such as FMT, particularly in immunocompromised patients, further delay clinical adoption. Finally, the absence of large, Randomized Controlled Trials and regulatory guidance makes it difficult to establish clinical recommendations. Overcoming these barriers will require multidisciplinary collaboration, the development of precision microbiome profiling tools, and the integration of microbiome science into personalized medicine frameworks.

## Figures and Tables

**Table 1:** Associations between specific microbial taxa, their effects on gene expression, and their role in colorectal oncogenesis [99].

Microorganism	Effects on Gene Expression	Effects on Colorectal Oncogenesis
*Fusobacterium nucleatum*	Increased abundance of *Fusobacterium nucleatum*—Downregulation of *Fc Receptor-Like A* (FCRLA), *Peptidase Inhibitor 16* (PI16), *Lymphocyte Specific Protein 1* (LSP1), and *Tumor Necrosis Factor Superfamily Member 9* (TNFSF9).	Facilitates immune evasion of tumor cells and promotes CRC progression.
*Prevotella* spp.	Decreased abundance of *Prevotella* spp.—Downregulation of *Metallothionein 1M* (MT1M).	May disrupt cellular defense mechanisms against CRC progression.
*Halomonadaceae*	Increased abundance of *Halomonadaceae*—Downregulation of *Reelin* (RELN).	May contribute to tumor invasion and poor prognosis in CRC patients.
*Paeniclostridium* spp.	Increased abundance of *Paeniclostridium* spp.—Upregulation of *Phospholipase C Beta 1* (PLCB1).	*PLCB1* is involved in cellular processes such as proliferation and apoptosis. The effect of increased *PLCB1* expression in CRC patients remains unclear.
*Enterococcus* spp.	Decreased abundance of *Enterococcus* spp.—Downregulation of *Immunoglobulin Superfamily Member 9* (IGSF9).	Facilitates immune evasion of tumor cells and promotes CRC progression.

**Table 2:** Protective bacterial species and their mechanisms against CRC.

Bacterial Species	Protective Mechanism Against CRC
*Lactobacillus* spp.	Modulates immune response: Stimulates IL-10 production, exerting anti-inflammatory effects, while inhibiting IL-6 and TNF-α production, thereby reducing pro-inflammatory signaling [174]. Anti-inflammatory effects and maintenance of intestinal homeostasis: Contributes to the regulation of immune responses and gut microbiota balance, preventing chronic inflammation [174]. Lactic acid production and intestinal pH reduction: Lowers intestinal pH, creating an unfavorable environment for pathogenic bacteria, thereby inhibiting their growth and colonization [175,176]. Production of SCFAs (acetate, butiyate, propionate) [177]. Degradation of carcinogenic agents: Reduces levels of N-nitrosamines, heterocyclic amines, and secondary bile acids [178,179,180].
*Bifidobacterium* spp.	Production of SCFAs, particularly butyrate: Reduces inflammation and induces apoptosis in tumor cells [181,182,183] Competition with procarcinogenic bacteria: Inhibits the growth of *Fusobacterium nucleatum* and *Bacteroides fragilis* [181,182,183]. Maintenance of intestinal barrier integrity: Strengthens tight junctions and enhances mucin production, preventing microbial translocation and inflammation [183].
*Faecalibacterium prausnitzii*	High butyrate production: The primary protective metabolite against cancer, exerting anti-tumor effects [171,184]. Anti-inflammatory effects: Stimulates IL-10 production and inhibits NF-κB signaling, reducing inflammation [171,184]. Reduction in oxidative stress and protection of epithelial cells: Mitigates cellular damage and maintains gut homeostasis [171,184].
*Akkermansia muciniphila*	Maintenance of the intestinal mucus layer: Protects against the translocation of pathogenic bacteria, preserving gut barrier integrity [185]. Regulation of host metabolism and inflammation: Enhances response to checkpoint inhibitor immunotherapy, contributing to better treatment outcomes [186]. Reduction in inflammation: Suppresses the production of pro-inflammatory cytokines [185].
*Roseburia* spp.	High butyrate production [187]. Anti-inflammatory effects [187]. Maintenance of intestinal barrier integrity: Strengthens tight junctions and preserves epithelial homeostasis, preventing bacterial translocation and inflammation [187]. Enhancement of response to immunotherapy (checkpoint inhibitors) [187].
*Faecalibaculum rodentium*	Beneficial effects on intestinal homeostasis and immune response [188]. Limited data on protective effects against CRC.
*Streptococcus thermophilus*	Probiotic properties [189]. Reduction in intestinal inflammation [189,190]. Limited data on protective effects against CRC.

**Table 3:** Current clinical evidence on gut microbiota modulation in CRC patients (RCTs—Randomized Controlled Trials, MSSmCRC—microsatellite stable metastatic colorectal cancer, FMT—fecal microbiota transplantation, dMMR—deficient mismatch repair) [193,194,195,196,197].

Study	Study Design	Intervention	Combined Therapy	Conclusions
Dikeocha et al., 2021 [193].	Systematic Review of 23 RCTs	Probiotics (*Lactobacillus*, *Bifidobacterium*)	Not specified	Probiotic supplementation improved quality of life, enhanced gut microbiota diversity, reduced postoperative infections, and inhibited pro-inflammatory cytokine production in CRC patients.
Zhao et al., 2023 [194].	Open-label, Single-arm, Phase II Trial (RENMIN-215)	FMT + Tislelizumab + Fruquintinib	Anti-PD-1 and anti-angiogenic therapy	Combination therapy showed improved survival with manageable safety in refractory MSS mCRC patients.
Zaharuddin et al., 2019 [195].	Open-label, Single-arm, Phase II Trial	Probiotics	Not specified	Probiotic supplementation reduced postoperative infections and improved gut microbiota composition in post-surgical CRC patients.
Yang et al., 2016 [196].	Randomized Controlled Trial	Perioperative Probiotics Treatment	Not specified	Probiotic supplementation after CRC surgery may exert anti-inflammatory effects by modulating the intestinal microenvironment.
MD Anderson Pilot Trial [197].	Open-label, Single-arm, Phase II Trial	FMT + Reintroduction of Anti-PD-1 Therapy (Pembrolizumab or Nivolumab)	Anti-PD-1 therapy	Study aims to assess whether FMT can enhance response to anti–PD-1 therapy in dMMR solid tumor patients; results pending.
